# A Qualitative Examination of Storage Practices of Women Firearm Owners in New Jersey and Ohio to Inform Suicide Prevention

**DOI:** 10.1016/j.focus.2025.100438

**Published:** 2025-09-10

**Authors:** Christina Rose Bauder, Jarrod M. Hay, Emma Unruh-Dawes, Sarah Millisor Irvin, Samantha E. Daruwala, Keelin G. Rademacher, Taylor Sabbagh, Craig J. Bryan, Kathryn A. Fassih

**Affiliations:** 1 Department of Psychiatry & Behavioral Health, The Ohio State University Wexner Medical Center, Columbus, Ohio; 2 The Center for the Advancement of Team Science, Analytics, and Systems Thinking in Health Services and Implementation Science Research (CATALYST), The Ohio State University Wexner Medical Center, Columbus, Ohio; 3 Department of Psychiatry, University of Vermont, Burlington, Vermont

**Keywords:** Firearms, women, qualitative, secure storage, suicide prevention

## Abstract

**Introduction:**

Firearms account for more than half of U.S. suicide deaths and nearly two thirds of all firearm-related deaths; although men are more likely to use a firearm than other lethal means, firearms account for approximately a third of suicide deaths for women. Suicide deaths involving firearms tend to be higher in states with fewer state firearm restrictions, with fewer suicide deaths involving firearms in states with more restrictive firearm laws. Critically, firearm storage practices are associated with an increased risk of both intentional and unintentional injury. The purpose of this study was to explore the experiences, attitudes, and practices of women firearm owners in New Jersey and Ohio to inform firearm-specific suicide prevention recommendations.

**Methods:**

The authors conducted semistructured qualitative interviews with 40-women firearm owners from New Jersey and Ohio between January and July of 2023. Transcripts were analyzed through thematic analysis using an inductive approach.

**Results:**

Nearly half of all participants (47.5%) reported at least 1 loaded firearm, with 50% reporting at least 1 unlocked firearm, and 11 (27.5%) reported at least 1 firearm that was both loaded and unlocked. Slightly fewer women reported storing firearms securely in New Jersey than in Ohio. The authors identified 5 common motivators in the storage practices of women, which included safety, upbringing and education, household composition, accessibility versus security, personal experiences of violence and victimization.

**Conclusions:**

Future research should explore unique opportunities to improve firearm risk communication and lethal means safety counseling among women and other underrepresented firearm owners to prevent intentional and unintentional firearm injury.

## HIGHLIGHTS

• Firearms are now the leading method for female suicide deaths in the U.S.
• States with stricter gun laws have fewer firearm-related suicides.
• Secure storage reduces suicide risk.
• Several key factors related to safety influence how women store their firearms.
• Suicide prevention strategies should be tailored to women gun owners.

## INTRODUCTION

Suicide remains a leading cause of death in the U.S., with preliminary estimates indicating just under 50,000 deaths in 2023.^1^^,^^2^ Of all suicide deaths, 6,623 deaths were of women.^2^ Firearms account for the largest proportion of suicide deaths; in 2022, firearms became the leading suicide method among women for the first time, surpassing poisoning and suffocation. Firearms account for approximately a third of female suicide deaths and are the leading method of suicide among female civilians and veterans.^1^^,^^3^ Increased focus on reducing firearm suicide risk has grown, given the high lethality of firearms compared with other lethal means.^4^ A recent meta-analysis confirmed firearms as the most lethal method of suicide, resulting in death in 90% of suicide attempts.^5^ Both ownership and access to firearms are risk factors for suicide, with the presence of firearms increasing the risk for both intentional and unintentional injury among all household members^6^, ^7^, ^8^ In a recent retrospective cohort study of adult women in California, suicide deaths by firearm increased after handgun acquisition by another adult household member.^9^ Storing firearms loaded, unlocked, or generally readily accessible is associated with^10^ increased risk of both intentional and unintentional injury.^11^

Approximately 32% of all adults in the U.S. are firearm owners, and around half of all new firearm owners from 2019 to 2021 were female.^12^^,^^13^ Female firearm ownership remains to be studied less often than other variations of ownership, despite the increasing rate of firearm-related suicides in women and approximately 42% of all firearm owners identifying as female.^14^, ^15^, ^16^, ^17^, ^18^, ^19^ Firearm access increases the risk of dying by suicide and homicide^20^^,^^21^ because approximately 54% of firearm-related deaths are suicide deaths; conversely, approximately 55% of all suicide deaths are by firearms.^14^, ^15^, ^16^, ^17^, ^18^^,^^20^ There are distinct differences between men and women around firearm ownership, reasons for ownership, access, and storage practices.^22^, ^23^, ^24^, ^25^ In comparison, women tend to obtain their first firearms at later ages, own fewer firearms, and are more likely to report owning handguns only than men.^26^ Women are also more likely to own firearms for protection, with 1 in 4 women and 1 in 5 men report owning a firearm for reasons pertaining to personal safety.^24^ Interpersonal violence experienced by women is among the leading reasons for this difference. Thompson and colleagues found that 44% of female participants aged 18–64 years reported at least 1 instance of physical, psychological, or sexual intimate partner violence during their lifetime.^27^ Exposure to interpersonal violence has been found to precede firearm acquisition, with some women reporting firearm ownership as a necessity for self-defense due to perceived gender-related vulnerabilities in the justice system.^24^^,^^28^^,^^29^ Paradoxically, increased access to firearms through ownership exponentially increases the risk that the firearm is used against someone in the household.^29^

There is an association between an increase in firearm suicide deaths in states with less restrictive gun laws and policies.^30^^,^^31^ Such examples include training required to purchase a firearm, firearm purchase waiting periods, and whether a permit for the concealed carry of a weapon is required.^32^ Results for a study using publicly available data, when compared with state-specific legislation, indicated that states with more prohibitive laws around the procurement and use of firearms had corresponding lower overall suicide rates and that a smaller proportion of suicides in such states resulted from firearms. Results also indicated that laws requiring registration and a license, such as in New Jersey, which requires a permit to purchase a firearm,^33^ had fewer suicide deaths overall as well as those involving firearms.^30^ Anestis and colleagues also found a decrease in overall suicide rates immediately after the implementation of laws requiring a license to own a handgun.^31^ Although legislative action is a larger ecologic prevention approach to reducing access to lethal means, such as increased waiting periods in the acquisition of a firearm, there are opportunities to reinforce prevention strategies at the individual and interpersonal levels through the promotion of secure storage practices.

Lethal means safety, which encourages secure storage of firearms, is one of the most promising ways of reducing suicide mortality.^17^^,^^34^^,^^35^ Secure firearm storage typically includes storing a firearm unloaded with a locking device or in a locked container, with ammunition stored separately. Examples of unsecure or unsafe storage practices typically include storing a firearm loaded, unlocked, and readily available (i.e., bedside table, on top of a piece of furniture). Evidence suggests that lethal means safety counseling can improve the use of secure storage practices; however, more research is needed to examine the effectiveness of available lethal means safety interventions.^36^

Less^14^^,^^17^ is known about potential key experiences, attitudes, and practices among women and other underrepresented firearm owners than among men. Potential distinctions may inform specific use and storage practices that lend insight to suicide prevention strategies such as secure storage practices, especially in relation to state-specific firearm laws.^14^^,^^15^^,^^17^ The overall goal of this study was to explore the experiences, attitudes, and practices of female firearm owners in New Jersey and Ohio—2 states with disparate firearm laws—to inform suicide prevention recommendations specific to secure storage of firearms (Table 1). The primary objective of the present analysis was to determine whether there are specific features of the experiences, attitudes, and practices of women firearm owners in both states that influence secure storage practices. The secondary objective is to explore possible differences in the experiences, attitudes, and practices of women firearm owners between New Jersey and Ohio on the basis of state residency and corresponding firearm laws and policies. The authors proposed 3 hypotheses: first, that most participants will report safe storage practices of unloading, locking, and securing firearms when not in use; second, that women will report a variety of motivating factors regarding safe storage practices; and third, that participants from Ohio, a state in which there are less restrictive firearm policies related to firearm control, firearm rights, and permit to carry, will report less safe storage practices than participants from New Jersey.

**Table 1:** Examples of State Gun Laws Between Ohio and New Jersey

Required law	Ohio	New Jersey
Background check and/or purchase permit		**X**
Concealed carry of a weapon permit required		**X**
Regulation of open carry		**X**
Extreme risk law		**X**
No carry after violent offense	**X**	**X**
Mental health prohibitor	**X**	**X**
Minimum age to purchase	**X**	**X**
Waiting periods		**X**

*Note*: These laws have been reviewed and verified as of January 15, 2025.

## METHODS

### Study Sample

Participants were eligible to participate if they identified as female, were aged at least 18 years, currently owned at least 1 handgun (e.g., pistol or revolver), and resided in either New Jersey or Ohio. Exclusion criteria included experiencing severe psychiatric symptoms or cognitive impairment precluding participation or imminent suicide risk warranting hospitalization. The authors screened 45 participants for eligibility, with 44 completing the baseline measures and 40 completing all study procedures, including the qualitative interview. The authors utilized quota sampling, in which the authors aimed to enroll an equal number of women from both New Jersey and Ohio.

### Procedures

The authors recruited participants using a combination of methods, including ResearchMatch, Facebook advertisements, and posting flyers on community boards in businesses (coffee shops, grocery stores, firearm retailers, and others) locally in central Ohio and northern New Jersey (Table 2). A copy of the recruitment flyer can be found in Appendix A (available online). Interested individuals were screened for eligibility through a virtual screening interview and asked questions about lifetime firearm ownership and practices. Those eligible were scheduled for a follow-up baseline interview with a research assistant, which included informed consent and assessment of sociodemographic and firearm ownership characteristics. Participants were then scheduled and participated in the qualitative interview at a separate time, lasting between 45 and 60 minutes. The interview guide can be found in Appendix B (available online). All research personnel were trained in crisis response planning for suicide prevention if any participants expressed suicide risk. A copy of the suicide risk management protocol is available by emailing the corresponding author. Participants received $60 for their participation in the form of an Amazon e-gift card. This study was approved by The Ohio State University IRB. Study data were collected and managed using REDCap (Research Electronic Data Capture) tools hosted at The Ohio State University.^37^

**Table 2:** Recruitment by Method

Strategy	*n* (%)
Facebook ads	23 (57.5)
Word of mouth	10 (25)
Research match	7 (17.5)

*Note*: Facebook advertisements were offered through The Ohio State University Center for Clinical and Translational Sciences.

### Measures

Demographic variables, including age, biological sex, gender identity, race, and ethnicity, in addition to firearm use and storage practices, were assessed through self-report survey. For firearm use, participants were asked to provide the age at which they acquired their first firearm, primary reason for owning a firearm, number of firearms currently in or around the home, and frequency of use/concealed carry. To assess firearm storage practices, participants were asked to identify their storage methods, locked/unlocked statuses, and loaded/unloaded statuses while they were stored.

To measure gun behaviors and beliefs, participants responded to items from the Gun Behaviors and Beliefs Scale (GBBS).^38^ The GBBS has 6 subscales: safety (i.e., Firearms protect people from criminals), emotional risk (i.e., Carrying a firearm makes me nervous), neighborhood concerns (i.e., The police frequently use firearms in my neighborhood), firearm presence (i.e., In my area, keeping firearms is an important tradition), social perceptions (i.e., I look up to people who carry a firearm), and firearm behaviors (i.e., I keep a loaded firearm at bedside). For 5 of the 6 subscales, individuals are asked to respond to a 7-point Likert scale response set (i.e., 1=strongly disagree to 7=strongly agree). The GBBS has demonstrated adequate internal consistency and reliability.^38^

A history of trauma was assessed using the Primary Care PTSD Screen for DSM-5.^39^ The Primary Care PTSD Screen for DSM-5 is a 5-item screener based on diagnostic criteria from the DSM-5 and addresses specific features of post-traumatic stress disorder (PTSD), specifically re-experiencing, hyperarousal, avoidance, and intrusion symptoms that have been experienced over the past month. The significance threshold for a higher likelihood of meeting criteria for PTSD is an endorsement of 3 or more items. Individuals who meet this threshold warrant further assessment for PTSD.

A semistructured qualitative interview was created to assess various constructs that included the timeline of firearm use and acquisition, firearm storage, firearms and identity, firearms and suicide, lethal means safety, and the influence of state legislation and policies on firearms ownership. Participants completed the interview with a member of the study staff who wrote down their responses and salient ideas that came from the conversation using REDCap.^37^ Interviews were completed by 4 members of the team, including the principal investigator; each member received training in foundational qualitative interviewing techniques and crisis response planning for suicide prevention as part of procedures to minimize risk.^40^ Interviews were audio and video recorded using an encrypted version of Microsoft Teams and were hand transcribed.

### Statistical Analysis

Thematic analysis was conducted using an inductive approach with assistance using ATLAS.ti (Version 23.2.1).^41^, ^42^, ^43^ All transcripts were independently reviewed by at least 2 members of the team and collectively reviewed among all members by analyzing and discussing constructed themes to work toward consensus (CRB, JH, SD, KR, and SI). Coders engaged in 2 cycles of coding. Focused coding entailed categorizing coded data on the basis of thematic or conceptual similarity.^44^ Team members used analytic memos to summarize responses to specific questions and constructs addressed in the interview, record salient themes and quotes, and describe impressions. Any notes that were taken during the interview were considered as part of the process of creating analytic memos. Transcript review and analysis recurred individually and iteratively, followed by ongoing, hourly, biweekly group meetings to discuss impressions and obtain consensus. Bracketing occurred initially and on an ongoing basis to acknowledge and address potential biases that could influence the process of interacting with the data.^45^ Team members discussed ways in which their identities, beliefs, and experiences could potentially influence their interpretations of the data; however, the authors acknowledged the value in their subjective interpretations as part of the coding and analytic process.

The authors used several strategies to improve the trustworthiness and credibility of their findings, beginning with a study rationale and introduction that preceded each interview (Appendix B, available online). The team was multidisciplinary, with a flattened power structure to ensure that all members had an equal opportunity to share perspectives and concerns throughout data collection and analysis. Study personnel who identified as women firearm owners, including the principal investigator, shared this during the introduction. Finally, the authors also triangulated the findings by reviewing responses to the eligibility interview, baseline questionnaires, and qualitative interview (i.e., investigator triangulation).^46^ After a series of discussions, accompanied by an iterative review of transcripts and analytic memos, the team agreed that consensus had been reached. The themes presented reflect those agreed upon after consensus. All quotes have been deidentified to protect participant anonymity.

## RESULTS

A complete table of participants' characteristics is available in Table 3, with firearm ownership characteristics presented in Table 4. Most women identified as Caucasian or White (*n*=39; 88.6%), with 5 participants identifying as African American or Black or Biracial (10.4%) (racial identity was collapsed to protect participants’ anonymity). Participants’ ages ranged from 25 to 73 years (mean=49.3 [SD=11.5] years). Slightly less than half (47.5%, *n*=19) of participants responded *yes* when asked whether any of their firearms were currently loaded; 50% reported that at least 1 firearm in the home was unlocked, and 20% reported that at least 1 firearm in the home was both loaded and unlocked. A total of 20 (50%) participants stored at least 1 firearm both unloaded and locked.

**Table 3:** Participant Characteristics

Characteristic	*n*=44	%
Gender, *n* (%)		
Female	44	100
Race, *n* (%)		
Caucasian or White	39	88.6
African American, Black, or Biracial	5	10.4
Age, years, mean (SD)	49.3	11.5

*Note*: Ethnicity value was omitted to preserve anonymity.

**Table 4:** Firearm Ownership Characteristics

Characteristic	*n*=44	%
Firearms in or around home, mean (SD)^a^		
Handguns	4.5	4.8
Shotguns	2.0	4.9
Rifles	2.3	3.4
Total	8.8	11.4
Reason for owning firearm, *n* (%)		
Personal safety or protection at home	26	59.1
Personal safety or protection away from home	2	4.5
Other recreational reasons	13	29.5
Competition	1	2.3
Other	2	4.5
Currently stored loaded, *n* (%)		
No	20	45.5
Yes	24	54.5
Currently stored unlocked, *n* (%)		
No	33	75.0
Yes	11	25.0
Currently stored loaded and unlocked, *n* (%)	9	20.5
Storage practices, *n* (%)		
Gun safe	31	70.5
Pelican case	11	25.0
Hidden in closet or drawer unloaded	7	15.9
Hidden in closet or drawer loaded	5	11.4
Gun cabinet	6	13.6
Locking device	4	9.1
Other	8	18.2
No storage methods	1	2.3
Planning to acquire in next 12 months		
No	22	50.0
Yes	14	31.8
Haven’t decided yet	8	18.2

a Outlier removed to preserve data quality. With outlier, results for firearms in or around home are handguns (mean=4.8, SD=11.6), shotguns (mean=4.9, SD=5.2), rifles (mean=3.4, SD=6.6), and total (mean=11.4, SD=21.4).

Gun safes were the most frequently reported storage practice (27; 67.5%), followed by pelican/hard cases (11; 27.5%). Seven (17.5%) participants reported hiding at least 1 firearm in a closet or drawer unloaded, with 5 (12.5%) reporting hiding at least 1 firearm in a closet or drawer loaded. Four participants each endorsed storing at least 1 firearm in a gun cabinet (10%) or using another locking device (10%), with 1 (2.5%) participant not using any storage practices.

There was no statistically significant difference between women in Ohio and New Jersey in endorsing the item asking, *Are any of your firearms loaded?*, with 21 (52.5%) women in Ohio and 19 (47.5%) in New Jersey responding *yes* (χ^2^=0.10, *p*=0.752). Of those who stored any firearms loaded (*n*=8; 20%), 5 women in Ohio (12.5%) and 3 (7.5%) women in New Jersey reported a firearm being both loaded and unlocked. Half of all participants (*n*=20; 50%) reported that at least 1 unloaded firearm was also unlocked, with 12 women (60%) from Ohio and 8 women (40 %) from New Jersey responding *yes*.

In the qualitative interviews, the authors asked questions eliciting further description of storage practices, access, and influencing factors on current storage practices (Appendix B, available online). Participants were asked, (1) *Who makes the primary decisions regarding firearm storage?* and (2) *How decisions around firearm storage were made in the household?* More than half of all participants (*n*=23, 57.5%) responded that they make the storage decisions in the home, with 11 (27.5%) saying that it was a joint decision with their spouse, 2 (5%) sharing that it was a decision between all adult members of the household, and 4 (10%) saying that their husband (all participants who reported a joint decision with a spouse identified them as their husband) made the decision.

Women were then asked whether they believed that there were differences in how they store their firearms on the basis of state-specific policies, such as provisions around conceal carry of a weapon or firearm purchasing requirements. One participant shared, “It has nothing to do with policy and everything to do with how I grew up,” with another, who had a family member in law enforcement stating, “[They’ taught me, ‘this is what you do… you store it in a certain way’, It was like, ‘This is the only way… if you're not willing to do it, you shouldn't own a gun.”

Women were then asked what additional factors might influence how they store their firearms. There were 5 prevailing themes influencing current storage practices that were constructed during the coding process, including safety, upbringing and education, household composition, accessibility versus security, and personal experiences of violence and/or victimization. Additional quotes under each theme are included in Table 5.

**Table 5:** Prevailing Themes and Corresponding Quotes Regarding Firearm Storage Practices

Theme	Quotes
Theme: Safety	• “Where people just get lazy and complacent, so that is something I'm extremely diligent about, because it's- it's easy to be lazy and complacent, and that's one thing they always go over in the trainings… they show videos of people you know, just getting home after the range and just being tired and throwing that range bag on the floor and just, God forbid, but it happens. And so those things have really impacted the way that I view the importance around knocking on my firearms and being diligent” (Ohio firearm owner, 30s) • “Personal protection from people and animals” (Ohio firearm owner, 40s)
Subtheme: Safety of children	• “It’s kind of a bounce back between home protection and personal protection… when I say home protection, it’s not about the stuff, it’s about the people” (Ohio firearm owner, 50s) • “If I had children, I would move the physical location to make it more inaccessible to a child” (Ohio firearm owner, 30s) • “My kids are very skilled with proper handling of firearms, but their friends may not be.” (Ohio firearm owner, 40s)
Subtheme: Safety of others in the household	• “It’s kind of a bounce back between home protection and personal protection… when I say home protection, it’s not about the stuff, it’s about the people” (Ohio firearm owner, 50s)
Theme: Upbringing and education	• “We never had guns just laying around [growing up], they’re always secured.” (New Jersey firearm owner, 50s) • “We grew up with firearms and that's how it was stored in a locked...closet” (New Jersey firearm owner, 40s) • “…it had everything to do with my upbringing.” (Ohio firearm owner, 60s)
Theme: Household composition	• “If there weren’t kids in the house, they’d be more accessible” (New Jersey firearm owner, 40s)
Theme: Accessibility versus security	• “I think if I didn’t have young children in the house, I would have them more accessible” (New Jersey firearm owner, 40s) • “I would like to have my protection firearms just a little more accessible if my life was threatened, we already store handguns locked up, but if I knew somebody who was injured inadvertently by handguns being in the house or not locked up, I might change” (Ohio firearm owner, 40s)
Theme: Personal experiences of violence and/or victimization	• “I was attacked by a [family member] … I felt unsafe… I needed some kind of protection when I was home alone” (New Jersey firearm owner, 40s). • “I've been mugged once many, many years ago, but our [city]... is seeing a major increase in drug use.” (Ohio firearm owner, 60s)

Participants shared safety as a primary influence in their current storage practices, with 2 subthemes including safety of children and safety of others in the household. Many participants emphasized the importance of preventing unauthorized access, especially by children or guests who may not be familiar with firearm safety. One participant shared, “The biggest thing is people getting injured, since I have kids. My kids are very skilled in [the] proper handling of firearms, but their friends may not be.” Another participant who had several children said, “…having children in the house, there’s natural curiosity for them to say, ‘look what my mom has!’, especially since we’re so open about shooting in the home.” Finally, one participant who had grown up in Southern Ohio with firearms responded that influencing factors around the storage of firearms included, “… how accessible they are to little hands. It also makes a difference in what I purchase, for example, I will find something that is harder to rack (the action of pulling the slide of a semi-automatic pistol back with your hand), they will have a hard time racking it and there's a safety”.

Many participants gave specific examples of their experiences growing up and the practices of their parents or other adults in their lives that informed the use and storage of firearms. Women shared how their orientation to firearms, whether during their upbringing or through other venues of education, influenced their storage practices. Most women (31 of 40) had taken a didactic course around firearms at some point in their lifetime, with 2 being trained firearms instructors. “We never had guns just laying around [growing up], they’re always secured,” one participant shared, with other participants similarly sharing expectations around firearms storage during their upbringing that informed how they made storage decisions in the present. Another shared, "We grew up with firearms and that's how it was stored in a locked...closet." Finally, one participant noted, “I grew up with guns my whole life… [specific storage] was just how I was raised.”

The authors identified household composition as an important theme in the current storage of firearms. Household composition also seemed to be tied to the theme of safety because many participants mentioned the presence (or absence) of children in the home as a motivating factor around specific storage practices. One participant cited, “If there weren’t kids in the house, they’d be more accessible,” when referring to the accessibility of the firearm dependent upon individuals who were in the home. One participant mentioned both household composition and safety as factors influencing storage, stating, “The biggest thing is people getting injured, since I have kids. My kids are very skilled in [the] proper handling of firearms, but their friends may not be.” Participants who did have children or lived in homes in which there were children spoke to the importance of keeping firearms out of reach of children, but several did speak to educating their children about any firearms or specifically indicating where and how they were stored.

Another participant’s response included the theme of accessibility versus security: “I don’t know, to be honest with you… I don’t want anyone who breaks in to have access to my guns, but then they would need to know that I have them, I wouldn’t want anyone to have access to them.” One participant shared, “We grew up with firearms… that’s how it was stored, locked…in a closet,” highlighting the influence of upbringing on storage practices. Another shared their experience growing up with firearms in the household, saying, “we never had guns just laying around the house, they were always secured.” Finally, one participant stated that a firearm for home defense was accessible in a specific area, where all other firearms were locked in a gun safe in the basement.

Finally, participants spoke to personal experiences around violence and/or victimization as they influenced their firearm use and storage practices. One participant from Ohio spoke to an incident involving an unknown person coming onto her property and attempting to open her door, stating, “…I keep one [round] in my chamber since that guy came around… a few seconds could make a difference.” Another spoke about an altercation that prompted the purchase of her first firearm, saying, “I was attacked by a [family member] … I felt unsafe… I needed some kind of protection when I was home alone,” and subsequent storage practices, which involved storing a loaded handgun in a locked biometric safe in an easily accessible location.

## DISCUSSION

The purpose of this study was to explore the experiences, attitudes, and practices of female firearm owners in New Jersey and Ohio—2 states with more and less restrictive firearm laws, respectively—to inform firearm suicide prevention recommendations, specifically the encouragement of secure storage practices. The results of this study provide novel information regarding the unique factors of women firearm owners because there has been limited qualitative examination of the experience of women firearm owners. Specifically, the authors examined 3 hypotheses: first, that most of the participants will report safe storage practices of unloading, locking, and securing firearms when not in use. The results showed that 47.5% of the sample reported storing any of their firearms currently loaded, 50% reported that at least 1 firearm in the home was unlocked, and 20% reported that at least 1 firearm in the home was both loaded and unlocked. These rates of firearm storage practices are partially reflective of firearm storage practices in the general population. Azrael and colleagues found in results from a survey assessing firearm storage in firearm-owning households with children that of these households, 21% stored at least 1 firearm loaded and unlocked, 50% stored at least 1 firearm with both loaded and locked or unloaded and unlocked, and 29% stored firearms both unloaded and locked.^47^ The results particularly align with those of prior work showing that half of individuals store at least 1 firearm loaded and that 20% of individuals store at least 1 firearm loaded and unlocked. These results replicate prior work in the sample of exclusively women, demonstrating that rates of certain firearm storage are reflective of other populations that might differ regarding demographic factors.

Second, the authors hypothesized that women would report different motivating factors regarding safe storage practices, specifically with regard to who and how decisions about firearm storage are made. Most of the participants disclosed that they were the ones to make the storage decisions in the home or that it was a joint decision made with their spouse. Participants also shared many different factors that motivate safe storage practices. Specifically, there were 5 themes that emerged, with safety as a primary influence on storage practices, and 2 subthemes emerged, including the safety of children and the safety of others in the household. Many participants emphasized the importance of preventing unauthorized access, especially by children or guests who may not be familiar with firearm safety. This result is very much reflective of the broader literature regarding storage practices, specifically that individuals are more likely to cite the safety of children and others in the home as a motivator for safe storage.^17^ Furthermore, prior work highlights that more women than men report this reason as a primary motivator for safe storage.^22^ The other themes that emerged consisted of upbringing and education, household composition, accessibility versus security, and personal experiences of victimization.

Finally, the authors hypothesized that women firearm owners in Ohio, a state in which there are fewer firearm policies related to gun control and stronger firearm-rights protections, will report poorer safe storage practices. The results showed that there was not a statistically significant difference between women in Ohio and those in New Jersey regarding storing firearms loaded. Of the 8 who stored any firearms loaded, 5 women in Ohio and 3 women in New Jersey reported a firearm being both loaded and unlocked. Half of all participants reported that at least 1 unloaded firearm was also unlocked, with a greater number being in Ohio. Importantly, these findings should be considered with caution given the small sample size.

The results of this study have implications for lethal means counseling and suicide prevention. Specifically, the results of this study emphasize the importance of approaching female firearm owners with lethal means safety counseling at the same frequency as male firearm owners.^17^^,^^48^ Spark and colleagues have highlighted the importance of tailoring lethal means counseling interventions to account for safety concerns as well as trauma lived experience.^17^^,^^49^ Given that several participants shared that victimization, either through intimate partner violence or through community violence, influenced their firearm use and storage, additional research should better anticipate. Although women are researched at a lower rate than men, they remain at risk for death by firearm suicide. The lack of research specifically on women means that ownership and storage-related factors as well as unique risk factors for firearm suicide are likely understudied. In fact, firearms account for approximately a third of female suicide deaths and are the leading method of suicide among female veterans,^1^ underscoring the importance of ongoing interventions for this population. Furthermore, although women tend to own fewer firearms than men, they are more likely to own handguns, which are more commonly used in suicide deaths than other types of firearms, including rifles and shotguns.^50^ This further emphasizes the importance of providing lethal means counseling interventions specifically for women.^51^

### Limitations

Several notable limitations exist in this study. First, the authors only recruited women living in New Jersey or Ohio, and although qualitative research is not fundamentally intended to be generalizable, the authors acknowledge the limitations in the applicability of these findings among women firearm owners in other states. In addition, although the authors utilized several strategies to reduce the influence of bias in interpreting transcripts through bracketing and triangulation and by involving women firearm owners on the study team, interpretations may be influenced by additional biases from the study team. Second, the sample was racially and ethnically homogeneous, which is not reflective of current women firearm owners. Furthermore, although purposeful sampling was undertaken, and the sample size was relatively large for a qualitative study, the small sample size, inductive approach, and qualitative interview design preclude generalizability to all women firearm owners. Finally, the authors did not report results of bivariate analyses given the underpowering for the last hypothesis; subsequent quantitative research will account for response rates across variables of interest through a priori power analysis.

## CONCLUSIONS

The findings of this study indicate several key considerations; women may not necessarily store firearms more securely than men, and a confluence of factors influences how and why women store their firearms in certain ways, including safety, upbringing and education, household composition, accessibility, personal experiences, and state policy. The results highlight the need for tailored lethal means counseling that addresses the specific concerns and experiences of female firearm owners, including priorities around the safety of self and others. As more women become first-time firearm owners, it is crucial to communicate safe storage practices and provide targeted interventions to reduce the risk of firearm-related suicides among women through firearms risk communication. Future research should further explore the factors influencing firearm storage behaviors among women and develop effective strategies to enhance firearm safety and prevent suicide. By understanding and addressing the unique needs of female firearm owners, the authors can make significant strides in reducing firearm-related injuries and deaths. Future research should highlight the unique lived experience of women firearm owners because they play a pivotal part in better understanding meaningful ways to improve firearm risk communication, increase lethal means safety through firearm secure storage practices, and reduce the number of lives impacted by suicide.

## CRediT authorship contribution statement

**Christina Rose Bauder:** Conceptualization, Data curation, Formal analysis, Funding acquisition, Investigation, Methodology, Project administration, Writing – original draft, Writing – review & editing. **Jarrod M. Hay:** Data curation, Investigation, Project administration, Visualization, Writing – original draft, Writing – review & editing. **Emma Unruh-Dawes:** Data curation, Formal analysis, Writing – original draft, Writing – review & editing. **Sarah Millisor Irvin:** Data curation, Investigation, Visualization, Writing – original draft, Writing – review & editing. **Samantha E. Daruwala:** Data curation, Investigation, Visualization, Writing – original draft, Writing – review & editing. **Keelin G. Rademacher:** Data curation, Investigation, Writing – original draft, Writing – review & editing. **Taylor Sabbagh:** Data curation, Visualization, Writing – original draft, Writing – review & editing. **Craig J. Bryan:** Conceptualization, Investigation, Supervision, Writing – original draft, Writing – review & editing. **Kathryn A. Fassih:** Writing – original draft, Writing – review & editing.

## Appendix Supplementary materials

Image, application 1

Image, application 2

## Footnotes

Supplementary material associated with this article can be found in the online version at doi:10.1016/j.focus.2025.100438.
