# Supplementary material for: A Qualitative Examination of Storage Practices of Women Firearm Owners in New Jersey and Ohio to Inform Suicide Prevention
Source: AJPM Focus. 2025 Sep 10;5(1):100438. doi: 10.1016/j.focus.2025.100438 (PMC12718134; doi:10.1016/j.focus.2025.100438)
Supplement: Supplementary file 1 [file mmc1.pdf]

Researchers at The Ohio State University are seeking women firearm owners residing in Ohio and New Jersey!

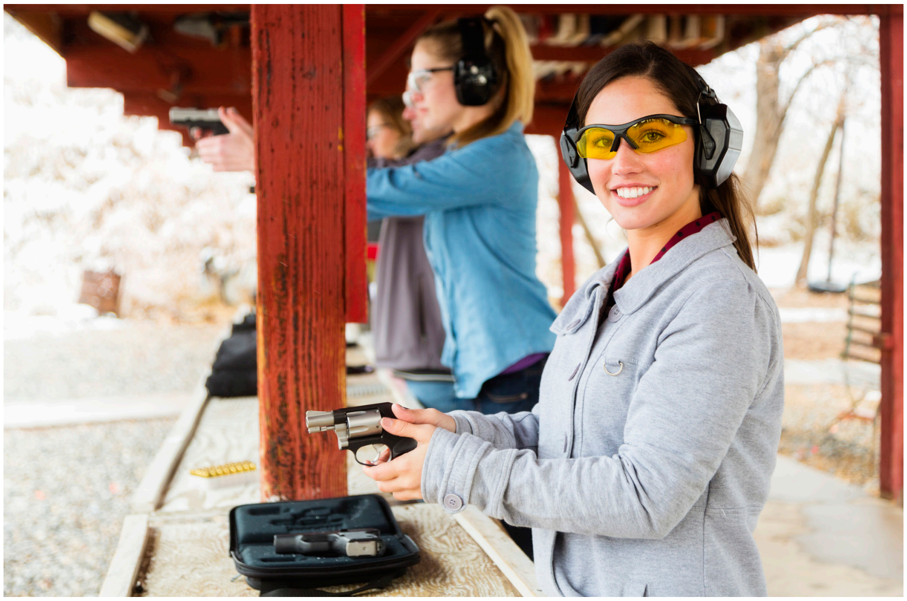

What is the purpose?

The purpose of this study is to explore the experiences, attitudes, and practices of female-identifying gun owners who live in either New Jersey or Ohio to inform firearm-specific suicide prevention practices. Eligible participants can receive $60 for participating.

Am I eligible?

- **Be at least 18 years old**
- **Identify as female**
- **Own at least one handgun**
- **Live in either New Jersey or Ohio**
- **Use an Internet-connected device with a webcam**

Scan the QR Code for more information!

THE OHIO STATE UNIVERSITY
WEXNER MEDICAL CENTER
