# Supplementary material for: A Qualitative Examination of Storage Practices of Women Firearm Owners in New Jersey and Ohio to Inform Suicide Prevention
Source: AJPM Focus. 2025 Sep 10;5(1):100438. doi: 10.1016/j.focus.2025.100438 (PMC12718134; doi:10.1016/j.focus.2025.100438)
Supplement: Supplementary file 2 [file mmc2.docx]

Exploring experiences, attitudes, and practices of firearms among women gun owners in New Jersey and Ohio

Interview Guide

Created September 7^th^, 2022

Updated September 12^th^, 2022

**Objectives**

The overall goal of this proposal is to explore the experiences, attitudes, and practices of female gun owners in New Jersey and Ohio– two states with more restrictive and less restrictive gun laws, respectively, – in order to inform firearm-specific suicide prevention recommendations**.** The **primary objective** of this project is to determine if there are specific features of the experiences, attitudes, and practices of women gun owners in both states that influence safe storage practices. The **secondary objective** is to explore possible associations among the experiences, attitudes, and practices of women gun owners based on state residency and corresponding firearm laws and policies. By focusing on these objectives, this project will likely confirm previous research on the diverse perspectives of stakeholders and expand upon potential key influences on gun use and safe storage practices from state firearm laws. To accomplish this objective, we will interview 40 participants in order to:

**Aim 1: To determine if there are specific features of the experiences, attitudes, and practices of women gun owners that influence safe storage practices.**

H1: We hypothesize that the majority of our participants will report safe storage practices of unloading, locking, and securing firearms when not in use.

H2: We hypothesize that women will report different motivating factors regarding safe storage practices.

**Aim 2: To explore possible associations among experiences, attitudes, and practices of women gun owners in New Jersey and Ohio and their state’s respective firearm policies.**

H3: We hypothesize that women gun owners in Ohio, a state in where there are less restrictive firearm policies related to gun-control, gun-rights, and permit to carry will report poorer safe storage practices.

H4: We hypothesize that women gun owners in New Jersey, a state in where there are more restrictive firearm policies related to gun-control, gun-rights, and permit to carry, will report stronger safe storage practices.

**Introduction and Warm Up**

*Thank you for your willingness to participate in our study. Before we begin, I’ll briefly introduce myself and describe the rationale behind the study and why we’re using hour-long interviews to answer our research questions.*

*My name is (****interviewer name****), and I’m a (****role name****) with The Ohio State University.*

*[Example of personal introduction] In addition to doing research around suicide prevention and firearms, I am also a gun owner. For me, there are a lot of factors that influence my identity as a gun owner, my practices around use and storage, when I carry versus don’t carry, and when I talk about guns with other people or tell them that I own guns (or don’t tell them).*

*Our goal with this study is to interview other women who own guns to learn more about their experiences, attitudes, and perspectives of firearms. We’re looking specifically at women gun owners in New Jersey and Ohio, because they have very different legislation and levels of restriction around guns. Ultimately, we can learn from these interviews to see if there are factors that might influence larger recommendations for use and storage practices as well as suicide prevention.*

*As a reminder, anything that you share here will be confidential. You have the right to refuse to respond to any of the questions that we discuss today. When we share the results of our study, we will take to steps to ensure that the information shared is not identifiable.*

- What’s something you’ve done recently that you’ve enjoyed?
- Tell me about how you found this study?
- What part/county of (Ohio/New Jersey) are you in?
- How long have you been in the area?
- How would you describe the area where you live (Urban, suburban, rural)?

**Gun Timeline**

*At this time, review the participant’s response to questions from the baseline survey around firearm ownership and acquisition.*

**Gun Use**

- What’s the primary reason you own a gun?
- How often do you use your handgun?
- If applicable, how often do you use the other guns you own, like rifles and shotguns?
- Do you conceal carry your firearm? If so, how often are you carrying your gun? Are there any times you won’t carry your gun?

**Gun Access**

- If you live with others, do other people in your home own guns?
- Who makes the primary decisions regarding storage, and how are decisions about firearm storage made in your household?
- Are there any children under the age of 18 in the household?
- Are they aware of where the guns are stored? Who else has access to your guns?

**Gun Storage**

- Can you give me an overview of how you store your guns?
- As part of our study, we are curious if there are differences in how women store their guns based on their experiences or the influences of state-specific policy. What do you think influences how you store your guns?
- Have you ever attended a firearms training course? Examples may be a CCW course before obtaining a CCW permit.
- Are there any circumstances in which you would change the way you store your firearms?
- Are there any circumstances in which you would ask someone to hold on to your guns? If so, who would you trust?
- When I mention the term firearms means safety, what comes to mind?
- What might be unique about women’s experiences, attitudes, and practices when compared to men?

**Guns and Identity**

- As you are likely aware, women are underrepresented as gun owners. What do you think influences low representation of women gun owners?
- Do you tell people that you own a gun or guns?
- Do you feel any certain way about identifying as a “gun owner”?
- Are you a member of any organizations marketed toward gun owners? Examples include shooting clubs, Girl with a Gun, the National Rifle Association, or membership organizations?

**Guns and Suicide**

- The overall goal of this study is to better understand experiences, attitudes, practices, of women gun owners to inform suicide prevention strategies. This is because over half of suicide deaths involve firearms.
  - What do you think is the connection between firearm storage and suicide?
- Each month in the United States, over 1,000 people die by suicide using firearms. Had a firearm not been accessible to them, how many do you think would have found another way to die by suicide? You can respond indicating the percentage from 0%-100% who you think would have found another way to die.
- What proportion of suicides do you consider preventable? Again, you can respond indicating the percentage from 0%-100%.
- Has suicide touched your life?

**Lethal Means Safety**

- Are you familiar with the term lethal means safety?
- If someone were going to speak with you about secure storage or lethal means safety, who would consider to be trustworthy? For example, would you trust a physician to talk to you about secure storage?
  - Would you trust a gun shop or range owner?
  - Would you be more receptive to the message if it came from a male gun owner or female gun owner?
  - How important is it that the source also identifies as a gun owner?

**Influence of State Legislation and Policies**

- Can you describe the extent to which you are familiar with your state’s gun laws? Some examples include a constitutional provision to keep and bear arms, the need for a permit to conceal carry a weapon.
- Are you familiar with policies around gun acquisition and permits to carry in other states?
- How would you rate the strength of your state’s gun laws?
- What would you change, if anything, about your state’s gun laws?
- Ohio- Recently, a bill was signed into permitting a qualifying adult to legally carry, possess, or conceal a handgun without a license, background check, or training requirements. How has this legislation impacted you?
- New Jersey- Recently, the Supreme Court ruled that New Jersians who wanted to apply for a permit to conceal carry no longer needs to demonstrate “justifiable need” to obtain such a permit. How has this legislation impacted you?

**Wrap Up**

- What questions or topics have we not covered in this interview?
- Is there anything you would like to add before we conclude?
